# Long-term efficacy and predictors of vagus nerve stimulation in drug-resistant epilepsy: a multicenter cohort study

**DOI:** 10.1186/s42494-025-00220-8

**Published:** 2025-09-01

**Authors:** Bojing Tan, Yuye Liu, Mingkun Gong, Fangang Meng, Anchao Yang, Kai Zhang, Lin Sang, Jianguo Zhang

**Affiliations:** 1 Department of Functional Neurosurgery, Beijing Tiantan Hospital, Capital Medical University, Beijing, 100070 China; 2 Beijing Neurosurgical Institute, Beijing, 100070 China; 3 Department of Neurosurgery, Capital Institute of Pediatrics, Beijing, 100020 China; 4 Department of Neurosurgery, Beijing Fengtai Hospital, Beijing, 100070 China

**Keywords:** Drug-resistant epilepsy, Vagus nerve stimulation, Predictor

## Abstract

**Background:**

At present, a number of indicators have been analyzed for the relationship with the efficacy of vagus nerve stimulation (VNS) in drug-resistant epilepsy (DRE) patients, but there is still no definite predictor of efficacy. This study is to assess the long-term effectiveness and predictors of VNS in DRE patients.

**Methods:**

We analyzed DRE patients monitored for over a year post-surgery (2016–2019) to evaluate VNS outcomes. Logistic regression was used to identify efficacy predictors.

**Results:**

Out of 162 DRE patients with VNS, 99 were followed for over 12 months, 80 for over 24 months, and 70 for over 36 months. At 12 months, 33 (33.4%) showed effectiveness, including 7 (7.1%) who were seizure-free. At 24 months, 32 (40.0%) were effective, including 12 (15.0%) who were seizure-free. At 36 months, 36 (51.4%) were effective, including 11 (15.7%) who were seizure-free. After 5 years, 27 (55.1%) were effective, including 8 (16.3%) who were seizure-free. Multivariate regression analysis identified structural etiology as a predictive factor for the effective VNS treatment (*P* = 0.039, OR = 0.35 [0.13–0.95]).

**Conclusions:**

VNS effectively controls seizures, with effectiveness and seizure-free rates improving over time. Patients with structural factors are at higher risk of ineffective VNS, suggesting epilepsy etiology may predict VNS outcomes.

## Background

Epilepsy is a common disease of the nervous system caused by different causes, with spontaneous, recurrent and unpredictable seizures as the main characteristics. It also has numerous neurobiological, cognitive, and psychosocial consequences. Drug therapy is the preferred treatment for epilepsy at present. Patients are usually first treated with anti-seizure medications (ASMs), but more than 30% of patients can not effectively control seizures after adequate trials of two tolerated and appropriately chosen ASMs. Epilepsy in these patients is defined as drug-resistant epilepsy (DRE) [1]. If drug therapy fails, seizures can also be controlled by surgery, neuromodulation and the ketogenic diet. Vagus nerve stimulation (VNS) is a form of neuromodulation therapy in which an electronic stimulator device is implanted in the neck and chest to stimulate the cervical vagus nerve to control seizures [2]. The common side effects of VNS mainly include hoarseness, cough, and paresthesia. Most of these side effects are temporary and transient. Adjusting the parameters of stimulation: current intensity, frequency, and duration can effectively reduce and alleviate these side effects in most patients.

It has been nearly 30 years since VNS was approved by FDA for the treatment of epilepsy in patients over 12 years of age in 1997. More than 130,000 patients with epilepsy have been treated with VNS worldwide. VNS devices made in China were approved for marketing in 2016, with more than 6000 epilepsy patients implanted with domestic VNS devices. The efficacy of VNS varies among DRE patients, and some respond poorly to treatment. Therefore, the screening of therapeutic indications is particularly important, and good screening of indications and preoperative evaluation may improve the efficiency and bring greater benefits to patients. In-depth analysis and study of the factors affecting VNS will not only help to find predictors of efficacy, but also may further reveal the mechanism of VNS in the treatment of epilepsy. Some patients experience social and mental health changes, with increased rates of anxiety, depression and sleep disorders [1, 2]. At the same time, regular adjustment of stimulation parameters and continuous effective follow-up become difficult. Remote control and remote follow-up provide the possibility to solve the dilemma. However, more medical institutions need to have the personnel and equipment to implement remote control and remote follow-up.

Therefore, we carried out this retrospective study to analyze the efficacy of VNS in the treatment of refractory epilepsy and its influencing factors, and to find predictors of efficacy.

## Methods

### Clinical data

DRE patients who underwent VNS stimulator implantation at Beijing Tiantan Hospital Affiliated to Capital Medical University and Beijing Fengtai Hospital from January 2016 to January 2019 and had follow-up data for more than one year were included in this study. Demographic characteristics, preoperative seizure frequency, scalp video-EEG, and MRI findings were collected using an electronic medical record system. During this period, the information of epileptic seizures and treatment of patients was obtained through interviews during outpatient or telephone follow-up. All patients or guardians gave informed consent and signed a written informed consent document before receiving VNS stimulator implantation for the possible use of their non-personal information for such medical research. Patients were informed of the study and informed consent was obtained in interviews after the study began.

### Inclusion and exclusion criteria

#### Inclusion criteria

①Seizures could not be controlled with reasonable and sufficient amount of ASMs, which met the definition of DRE by the International League Against Epilepsy (International League Against Epilepsy, ILAE); ②According to multidisciplinary preoperative evaluation, resection surgery is not suitable, or previous craniotomy epilepsy surgery is performed; ③According to multidisciplinary preoperative evaluation, VNS stimulator implantation is suitable; ④Postoperative follow-up time is at least 1 year.

#### Exclusion criteria

①The preoperative medical records and auxiliary examination results were incomplete; ②VNS stimulator was taken out after operation; ③Underwent other surgical operations after implantation; ④Loss of follow-up after operation; ⑤Died; ⑥Unable to continue to complete follow-up and related tests according to the requirements of the study.

### Seizure semiology and etiology

Seizure semiology variables were classified based on scalp video-EEG recordings, patient's subjective experiences, and witness descriptions, according to the 2017 ILAE [3] and 2019 Lüders et al. [4] classification systems, including seizure types like aura, autonomic, atonic, stupefied, epileptic, focal tonic, focal clonic, automotor, and focal to generalized tonic–clonic seizures. Impaired consciousness during seizures was also considered. The epilepsy etiology was determined based on seizure manifestations, imaging (MRI, PET/CT, MEG), genetic tests, electrophysiology, and blood/urine laboratory analyses.

### VNS stimulator implantation surgery

Under general anesthesia, a Chinese PINS vagus nerve pulse generator (G111 for adults, G112 for children) was implanted on the left vagus nerve in all patients. The patient was positioned supine with the head tilted right to expose the left neck. The procedure involved making incisions in the neck and chest. A 4-cm incision was made 3 to 5 cm below the left clavicle to create a pocket for the pulse generator by separating the pectoralis major fascia and fat. In some younger children, the pocket was made under the fascia. A 3-cm neck incision extended from the left mandibular angle to the distal 2/3 of the clavicle, along the anterior border of the sternocleidomastoid muscle. The deep cervical fascia was opened to expose and incise the carotid sheath at the inferior border of the omohyoid muscle. Once the vagus nerve is exposed, electrodes are carefully wrapped with the nerve. Finally, a subcutaneous tunnel is created between the neck and chest to connect the electrode to the pulse generator.

### Stimulation parameter adjustment

Two weeks post-operation, the stimulator was activated with initial settings of 0.20 mA current, 500 μs pulse width, 30 Hz frequency, 30 s stimulation time, and 5 min intermittent time. The current was increased by 0.1–0.3 mA every two weeks, while other settings remained constant. Adjustments were made either during clinic visits, where an epilepsy specialist assessed and adjusted parameters based on the patient's condition, or through remote video diagnosis and program-controlled adjustments. After three months, the VNS treatment effect was evaluated, and if stable, the adjustment intervals were extended based on seizure frequency.

### Follow-up and efficacy evaluation

Prior to VNS stimulator implantation, the patient was instructed to return to the epilepsy clinic for review and adjustment of stimulation parameters at 0.25, 0.5, 1 year, and annually thereafter. Follow-up included seizure-related conditions (seizure frequency, symptoms, severity, duration), use of ASMs, and VNS-related complications. At other time points, patients may voluntarily report their postoperative condition and receive in-person or remote programming.

At 1 year after VNS device implantation, if a patient has a decrease in seizure frequency by 50% or more, the VNS treatment is considered effective (responder), including patients who were seizure-free after VNS stimulator implantation. VNS treatment was considered to be non-responder if the seizure frequency of the patient was reduced by ≤ 50%.

### Statistical analysis

A descriptive summary of baseline and relevant clinical data was generated to characterize the study population. Kolmogorov–Smirnov test was used to determine whether continuous variables were normally distributed. Continuous variables were described by mean ± standard deviation (mean ± SD) if they were normally distributed and by median and interquartile range (median, [IQR: P25, P75]) if they were not normally distributed. Categorical variables were described by frequency and percentage (%).

Continuous variables conforming to normal distribution were compared by *t*-test, and continuous variables not conforming to normal distribution were compared by nonparametric test. Chi-square test or Fisher's exact test was used to compare the categorical data. Clinical variables associated with postoperative seizures were analyzed by univariate and multivariate logistic regression. *P* < 0.05 was considered statistically significant. Clinical variables associated with the quality-of-life score were analyzed by linear regression. Data reduction and statistical analysis were performed using R studio 4.3.2.

## Results

### Clinical characteristics

Ninety-nine patients who underwent VNS stimulator implantation for more than one year in Beijing Temple of Heaven Hospital and Beijing Fengtai Hospital affiliated to Capital University of Medical Sciences from January 2016 to January 2019 were included in this study, including 71 males and 28 females, with an average age of 15.72 ± 10.22 years at the time of operation. The detailed clinical characteristics of patients treated with VNS are shown in Table 1.

**Table 1:** Characteristics of patients

Variables	Total	Non-responder	Responder	Statistic	*P*
	(*n* = 99)	(*n* = 66)	(*n* = 33)		
**Demographics and disease history**					
Age at surgery, Mean ± SD	15.72 ± 10.22	15.20 ± 10.26	16.76 ± 10.21	t = −0.71	0.479
Age at seizure onset, Mean ± SD	6.68 ± 7.26	6.43 ± 7.58	7.17 ± 6.67	t = −0.48	0.632
Seizure duration, Mean ± SD	8.77 ± 7.09	8.32 ± 7.08	9.64 ± 7.13	t = −0.87	0.386
Gender, *n* (%)				χ^2^ = 1.22	0.269
Male	71 (71.72)	45 (68.18)	26 (78.79)		
Female	28 (28.28)	21 (31.82)	7 (21.21)		
Previous disease history, *n *(%)				χ^2^ = 2.56	0.278
Single	31 (31.31)	21 (31.82)	10 (30.30)		
Multiple	11 (11.11)	5 (7.58)	6 (18.18)		
No exact	57 (57.58)	40 (60.61)	17 (51.52)		
Intracranial hemorrhage, *n *(%)	3 (3.03)	1 (1.52)	2 (6.06)	χ^2^ = 0.39	0.534
HIE, *n *(%)	14 (14.14)	8 (12.12)	6 (18.18)	χ^2^ = 0.26	0.61
Craniocerebral trauma, *n *(%)	8 (8.08)	5 (7.58)	3 (9.09)	χ^2^ = 0.00	1
Craniotomy, *n *(%)	6 (6.06)	2 (3.03)	4 (12.12)	χ^2^ = 1.80	0.18
Intracranial infection, *n *(%)	13 (13.13)	9 (13.64)	4 (12.12)	χ^2^ = 0.00	1
Febrile seizure, *n *(%)	9 (9.09)	6 (9.09)	3 (9.09)	χ^2^ = 0.00	1
Cerebral palsy, *n *(%)	2 (2.02)	1 (1.52)	1 (3.03)	-	1
**Seizure semiology**					
Aura, *n *(%)	13 (13.13)	7 (10.61)	6 (18.18)	-	0.569
Autonomic seizure, *n *(%)	5 (5.05)	3 (4.55)	2 (6.06)	-	1
Atonic seizure, *n *(%)	15 (15.15)	12 (18.18)	3 (9.09)	-	0.59
Dialeptic seizure, *n *(%)	25 (25.25)	19 (28.79)	6 (18.18)	-	0.587
Epileptic spasm, *n *(%)	16 (16.16)	14 (21.21)	2 (6.06)	χ^2^ = 3.73	0.054
Impaired aware, *n *(%)	72 (72.73)	44 (66.67)	28 (84.85)	-	0.111
GTCS,* n* (%)	40 (40.40)	24 (36.36)	16 (48.48)	-	0.533
Tonic seizure, *n *(%)	37 (37.37)	27 (40.91)	10 (30.30)	-	0.619
Clonic seizure, *n *(%)	20 (20.20)	15 (22.73)	5 (15.15)	-	0.745
Automatisms, *n *(%)	22 (22.22)	17 (25.76)	5 (15.15)	-	0.573
Multiple forms of seizures, *n *(%)	34 (34.34)	25 (37.88)	9 (27.27)	-	0.614
**MRI,** ***n*** **(%)**				χ^2^ = 0.36	0.836
Negative	20 (20.20)	14 (21.21)	6 (18.18)		
Positive	41 (41.41)	28 (42.42)	13 (39.39)		
Uncertain or absent	38 (38.38)	24 (36.36)	14 (42.42)		
**Etiology**					
Etiological complexity, *n *(%)				χ^2^ = 0.02	0.89
Single	92 (92.93)	62 (93.94)	30 (90.91)		
Multiple	7 (7.07)	4 (6.06)	3 (9.09)		
Structural, *n *(%)	46 (46.46)	34 (51.52)	12 (36.36)	χ^2^ = 2.03	0.154
Infectious, *n *(%)	15 (15.15)	9 (13.64)	6 (18.18)	χ^2^ = 0.35	0.552
Genetic, *n *(%)	1 (1.01)	0 (0.00)	1 (3.03)	-	0.333
Metabolic, *n *(%)	1 (1.01)	0 (0.00)	1 (3.03)	-	0.333
Unknown, *n *(%)	42 (42.42)	27 (40.91)	15 (45.45)	χ^2^ = 0.19	0.666

*Abbreviations:* *HIE* Hypoxic-ischemic encephalopathy, *GTCS* Generalized tonic–clonic seizure

### Efficacy of VNS

**Fig. 1:**
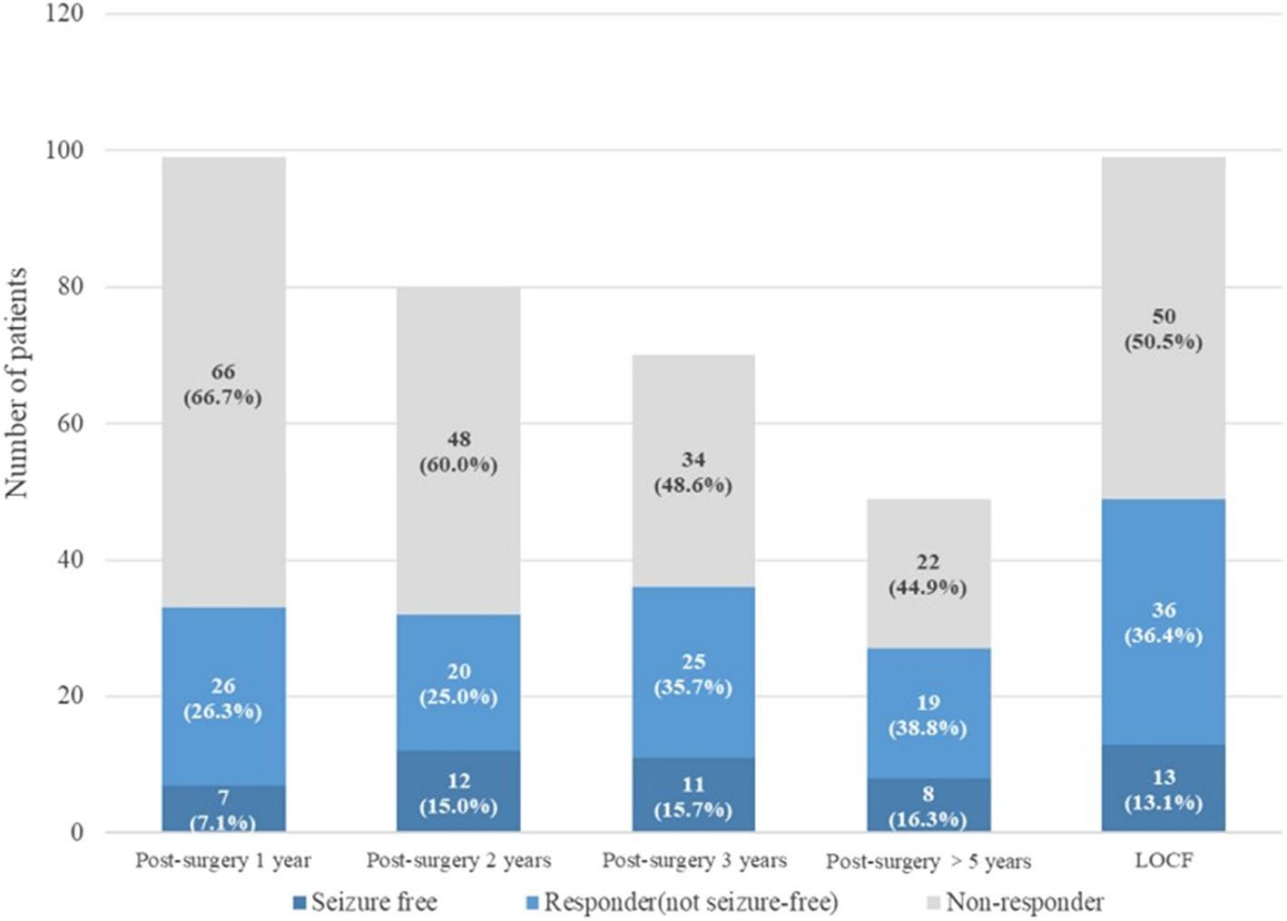
Patients responded to VNS over time. LOCF: Last observation carried forward

**Fig. 2:**
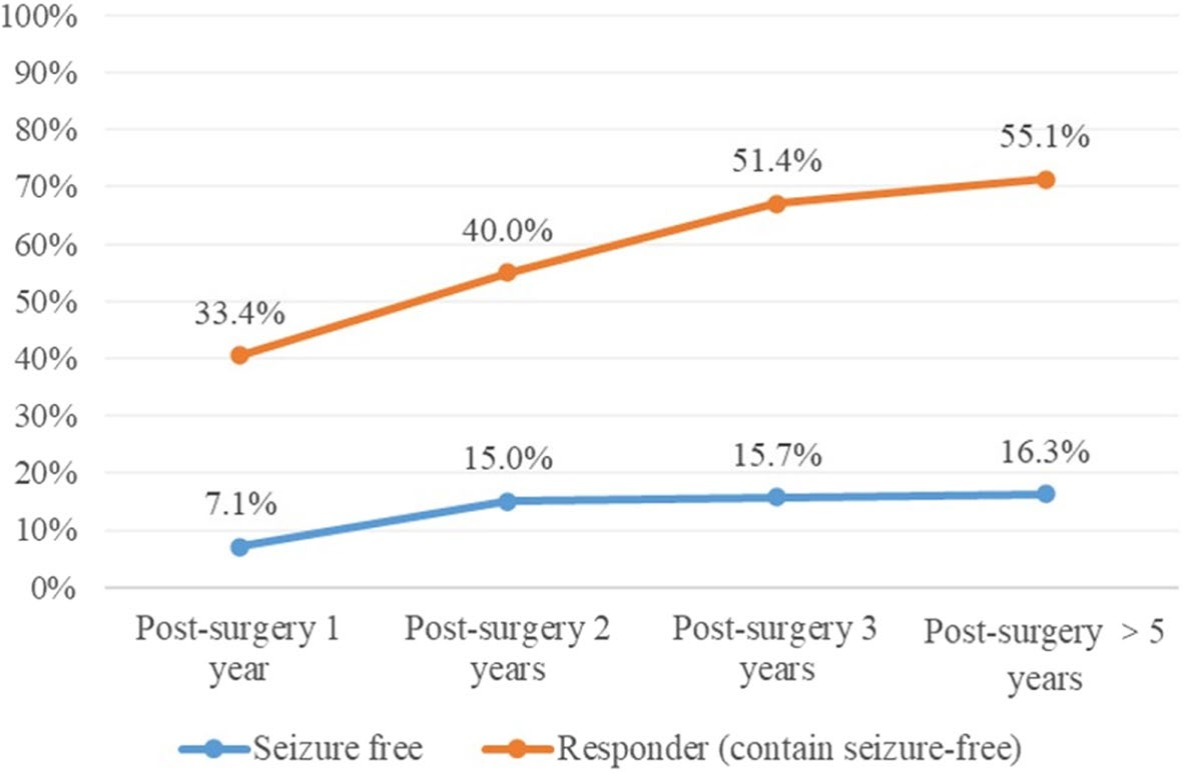
Seizure outcomes in long-term efficacy of VNS

Among the 99 patients treated with VNS for more than 1 year, 80 continued treatment for more than 2 years and 70 for more than 3 years. At 1 year after VNS device implantation, 33 (33.4%) patients responded to treatment, of whom 7 (7.1%) were seizure-free. For the 80 patients treated with VNS for more than 2 years, 32 (40.0%) responded to treatment by the 2-year follow-up, with 12 (15.0%) achieving seizure-freedom. Similarly, among the 70 patients treated with VNS for more than 3 years, 36 (51.4%) responded to treatment, including 11 (15.7%) were seizure-free cases. As for the 49 patients, with continuous VNS treatment and follow-up for more than 5 years, 27 (55.1%) responded to treatment, with 8 (16.3%) were seizure-free (Figs. 1 and 2).

In addition, different patients successfully completed the last follow-up at different times, and some were only more than one year after operation. Thus, of the 99 patients who completed the last follow-up visit, 49 responded to treatment and 13 were seizure-free. The effective rate was only 49.5%, and the seizure-free rate was 13.1%, both of which were lower than follow-up rate in 5 years.

### Influencing factors of outcome

Univariate logistic regression analysis found no statistically significant association between clinical variables and the efficacy of VNS. Five clinical variable was included in the multivariate regression analysis. Multivariate regression analysis showed that structural etiology was a protective factor for the effectiveness of VNS [*P* = 0. 039, OR = 0. 35 (0. 13–0. 95)] (Table 2).

**Table 2:** Univariate and multivariate logistic regression analysis

Variables	Univariate					Multivariate				
	β	S.E	Z	*P*	OR (95%CI)	β	S.E	Z	*P*	OR (95%CI)
**Demographics and disease history**										
Female	−0.55	0.5	−1.1	0.272						
Age at seizure onset	0.01	0.03	0.48	0.63	1.01 (0.96, 1.07)					
Previous disease history, *n* (%)										
Single					1.00 (Reference)					1.00 (Reference)
Multiple	0.92	0.72	1.29	0.197	2.52 (0.62, 10.28)	0.9	0.82	1.09	0.274	2.46 (0.49, 12.39)
No exact	−0.11	0.48	−0.24	0.813	0.89 (0.35, 2.29)	0.07	0.53	0.13	0.899	1.07 (0.38, 3.02)
Seizure duration	0.03	0.03	0.87	0.384	1.03 (0.97, 1.09)					
Intracranial hemorrhage	1.43	1.24	1.15	0.249	4.19 (0.37, 48.03)					
HIE	0.48	0.59	0.81	0.417	1.61 (0.51, 5.10)					
Craniocerebral trauma	0.2	0.76	0.26	0.795	1.22 (0.27, 5.45)					
Craniotomy	1.48	0.89	1.66	0.097	4.41 (0.76, 25.48)	1.73	1.08	1.61	0.108	5.64 (0.68, 46.62)
Intracranial infection,	−0.14	0.64	−0.21	0.833	0.87 (0.25, 3.08)					
Febrile seizure	0.71	1.43	0.5	0.62	2.03 (0.12, 33.53)					
Cerebral palsy	0	0.74	0	1	1.00 (0.23, 4.28)					
**Seizure semiology**										
Multiple forms of seizures,	−0.49	0.47	−1.05	0.293	0.61 (0.24, 1.53)					
Aura	0.63	0.6	1.04	0.297	1.88 (0.57, 6.15)					
Autonomic seizure	0.3	0.94	0.32	0.746	1.36 (0.21, 8.55)					
Dialeptic seizure	−0.6	0.53	−1.14	0.254	0.55 (0.19, 1.54)					
Atonic seizure	−0.8	0.69	−1.17	0.242	0.45 (0.12, 1.72)					
Epileptic spasm	−1.43	0.79	−1.81	0.07	0.24 (0.05, 1.13)	−1.02	0.89	−1.15	0.25	0.36 (0.06, 2.06)
Impaired aware	1.16	0.6	1.93	0.053	3.18 (0.98, 10.29)	0.94	0.67	1.4	0.161	2.57 (0.69, 9.60)
GTCS	0.51	0.44	1.17	0.243	1.67 (0.71, 3.93)					
Focal tonic seizure	−0.47	0.46	−1.03	0.301	0.62 (0.25, 1.53)					
Focal clonic seizure	−0.5	0.57	−0.88	0.377	0.60 (0.20, 1.85)					
Automatisms	−0.67	0.56	−1.19	0.235	0.51 (0.17, 1.54)					
**Etiology**										
Multiple causes	0.44	0.8	0.55	0.582	1.55 (0.33, 7.37)					
**Structural**	−0.62	0.44	−1.42	0.157	0.54 (0.23, 1.27)	−1.06	0.51	−2.07	**0.039**	0.35 (0.13, 0.95)
Infectious	0.34	0.58	0.59	0.553	1.41 (0.45, 4.36)					
Genetic	16.29	1455.4	0.01	0.991	11,875,489.02 (0.00, Inf)					
Metabolic	16.29	1455.4	0.01	0.991	11,875,489.02 (0.00, Inf)					
Unknown	0.19	0.43	0.43	0.666	1.20 (0.52, 2.80)					
**MRI**										
Negative					1.00 (Reference)					
Positive	0.08	0.59	0.14	0.892	1.08 (0.34, 3.46)					
Uncertain or absent	0.31	0.59	0.52	0.603	1.36 (0.43, 4.35)					

*Abbreviations:* *HIE* Hypoxic-ischemic encephalopathy, *GTCS* Generalized tonic–clonic seizure

## Discussion

Differences in morbidities, age at onset, epilepsy duration, and seizure type lead to varied outcomes with VNS treatment. Therefore, a thoroughly examination of factors affecting VNS efficacy is crucial. Identifying these factors can help find reliable predictors of treatment response, improve preoperative evaluations, and select suitable VNS candidates. This study seeks to assess VNS's clinical effectiveness in managing drug-resistant epilepsy and identify potential predictors of treatment response.

### Overall efficacy after VNS

VNS efficacy for DRE is primarily measured by seizure control, EEG changes, ASM use, and quality of life improvements. Seizure reduction is the main goal. While the Engel [5] and ILAE classification systems [6] are common, they have limitations for VNS assessment. The McHugh scale, introduced in 2007 [7], is now preferred in clinical practice for its practicality. A seizure frequency reduction of over 50%, aligning with McHugh grade I or II, is a key indicator of successful VNS treatment.

While the efficacy of VNS requires further confirmation, four double-blind, randomized, controlled trials have compared pre- and post-operative efficacy of VNS using low-parameter stimulation with theoretically no therapeutic effect versus conventional parameter stimulation [8–11]. At the end of the 14- to 20-week double-blind periods, three studies reported statistically significant between group differences in seizure frequency reduction from baseline [8–10]. There was no statistically significant difference in the proportion of children between the study groups [11]. When evaluating >50% seizure reduction, two studies showed statistically significant inter-group differences. Meta-analyses of these RCTs revealed that high-intensity stimulation was more likely to achieve a reduction in seizure frequency than low-intensity stimulation in adults (>50% seizure reduction: *P* = 0.01, OR = 1.95 [1.16–3.27]); in pediatric patients, but this effect did not reach statistical significance in pediatric cohorts (*P* = 0.73, OR = 0.75 [0.14–3.90]) [12, 13].

Several uncontrolled studies have looked at long-term outcomes after VNS in children and adults and have shown that patients who underwent VNS have sustained benefits with an overall response rat. VNS treatment of pediatric patients not only helps some patients to reduce seizures, but also significantly improves cognitive function, such as enhanced intelligence, attention, better memory and language ability. Long-term efficacy analysis, especially some studies with follow-up of more than 5 years, shows that VNS treatment may be increasingly effective over time, with response rates reaching 60–70% [14, 15]. In a study of data from the VNS Treatment Effectiveness Registry in the United States, 5554 patients with epilepsy were enrolled. At 2–4 years post-VNS, 63% of the patients were treated effectively, including an 8.2% seizure-free rate. The response rate and sustained seizure-free rate increased gradually over time [16]. However, the design of these studies may obscure the impact of ASM modifications on VNS efficacy. In a prospective study, there was no statistically significant difference in the response rate to VNS between patients who maintained stable ASM regimens during VNS therapy and those who were allowed to change their ASMs. Importantly, patients who avoided ASM changes after VNS continued to benefit [17].

Our study demonstrated a 5-year response rate of 55.1%, with a seizure-free rate of 16.3%, slightly lower than rates reported in some previous studies. This may be due to the higher proportion of adults in our cohort, consistent with evidence suggesting relatively lower VNS efficacy in adults versus pediatric populations, while the moderate sample size could introduce selection bias.

### Predictors of VNS efficacy

VNS is effective for DRE, but shows considerable interindividual variability. Different from Class I and Class II evidence, there remains a lack of high-grade evidence regarding predictors of VNS response, and most existing studies are single-center investigations with limited sample sizes and lack of controls. With the development of genetics, imaging, autoimmune-associated antibody detection and bioinformatics, our understanding of the etiology of epilepsy has gradually deepened. In 2017, the ILAE Classification Working Group reclassified epilepsy etiologies into six categories: genetic, structural, metabolic, immune, infectious and unknown [3].

Multivariate regression analysis showed that structural etiology was a predictive factor for the effectiveness of VNS, and patients with structural etiology had a higher risk of VNS failure. Assessing structural factors could guide clinical decision-making, facilitating patient selection and treatment optimization. However, further analysis of unilateral and bilateral structural abnormalities showed no significant difference, suggesting that further exploration is needed to determine which structural factors are more strongly associated with efficacy. A meta-analysis of 78 VNS studies suggests that the etiology of epilepsy may be a predictor of postoperative seizure freedom [16], which is similar to this study. Theoretically, exploring the common points between the epilepsy etiology and the VNS mechanism could help clarify their relationship with the efficacy of VNS, but such studies remain limited. On the other hand, according to the different classification of epileptic seizure semiology, such as seizure aura, autonomic seizure, atonic seizure, stupefied seizure, epileptic spasm, impaired consciousness, GTCS, tonic seizure, clonic seizure, automotor seizure, regression analysis revealed no statistical difference [3, 4]. Englot et al. [18] pointed out that the effective rate and seizure reduction rate of post-traumatic epilepsy patients treated with VNS are significantly higher than those of non-post-traumatic epilepsy patients, and compared with generalized tonic–clonic seizures, tonic seizures have a better prognosis, which may indicate that there is still a long way to go to explore the predictive indicators of efficacy. It is worth noting that no statistical difference in the efficacy of patients after brain surgery, which may suggest that VNS is still a viable treatment for those ineligible for resection or with persistent post-surgical seizures.

In theory, early implantation of VNS devices for neuromodulation therapy can reduce seizures by activating the corresponding brain structures and regulating the release of neurotransmitters during the critical period of brain development in children, and ultimately prevent both seizure-induced brain damage and abnormal circuit formation. Patient age at surgery has been a predictor of VNS efficacy of interest to investigators, although previous studies have shown no significant association between implantation age and efficacy [19, 20]. However, some studies have shown that even if there is no significant difference in the effective rate, younger patients (< 5 years) have higher responder rates than older adolescents (> 15 years) indicating earlier intervention may yield greater clinical benefits [21].

Similarly, the impact of epilepsy duration on VNS efficacy is still uncertain. A single-center study of 77 patients stratified by disease course (using 12.5 years as a cutoff) found significantly higher response rates in patients with shorter disease duration (<12.5 years), suggesting earlier VNS intervention may yield superior outcomes [22]. Russo et al. [23] also pointed out that pediatric patients who underwent VNS implantation within 3 years of epilepsy exhibited the highest response rates, while those implantated between 3 and 5 years after epilepsy onset demonstrated better long-term seizure freedom. Helmers et al. [24] reported that the proportion of patients who achieved seizure-freedom with VNS was significantly higher in those with epilepsy duration ≤6 years compared to those with longer disease duration. A study conducted by Englot et al. [16] including 5554 cases further supported this finding, demonstrating a higher response rate in patients treated within 10 years of epilepsy onset. In contrast, Renfore et al. [25] analyzed 2785 cases with disease duration > 5 years against 120 cases with shorter duration, but found no significant difference in the efficacy of VNS—including median seizure reduction rates or the proportion of patients achieving ≥ 50% seizure reduction.

### Limitations

This study has several limitations. Despite the data being sourced from two epilepsy centers, the uniformity in diagnostic and therapeutic approaches, as well as clinical techniques across these centers, may introduce potential selection and information biases, potentially affecting the data's accuracy. Future research should seek to validate our findings by increasing the sample size and incorporating a broader range of centers. Furthermore, beyond the fundamental clinical characteristics of patients, there is a growing interest among researchers in identifying biomarkers that could predict the efficacy of VNS through neuroimaging or electrophysiological assessments.

## Conclusions

VNS has demonstrated efficacy in managing DRE. Longitudinally, the efficacy and seizure-free rates associated with VNS treatment tend to improve over time. Specifically, at the 5-year follow-up, the efficacy and seizure-free rates are reported to be 55.1% and 16.3%, respectively. However, the etiology of epilepsy may serve as a prognostic indicator of VNS success, patients with structural factors have a higher risk of VNS failure. It is advisable for clinicians to cautiously and thoroughly evaluate the potential effectiveness of VNS in patients with a confirmed structural etiology of epilepsy.

## Data Availability

The datasets used and/or analyzed during the current study are available from the corresponding author on reasonable request.

## Abbreviations

ASMs: Anti-seizure medications
DRE: Drug-resistant epilepsy
EEG: Electroencephalogram
GTCS: Generalized tonic–clonic seizure
ILAE: International League Against Epilepsy
MEG: Magnetoencephalography
MRI: Magnetic resonance imaging
PET/CT: Positron emission tomography/Computed tomography
RCT: Randomized controlled trial
SD: Standard Deviation
SUDEP: Sudden unexpected death in epilepsy
VNS: Vagus nerve stimulation
